# We Need Engaged Workers! A Structural Equation Modeling Study from the Positive Organizational Psychology in Times of COVID-19 in Chile

**DOI:** 10.3390/ijerph19137700

**Published:** 2022-06-23

**Authors:** Rodolfo Mendoza-Llanos, Álvaro Acuña-Hormazábal, Olga Pons-Peregort

**Affiliations:** 1 Social Sciences Department, Universidad del Bío-Bío, Chillán 3800708, Chile; rmendoza@ubiobio.cl; 2 Business Management Department, Universidad del Bío-Bío, Chillán 3800708, Chile; 3 Department of Business Organization, Universidad Politécnica de Catalunya, 08034 Barcelona, Spain; olga.pons@upc.edu

**Keywords:** engagement, burnout, healthy organizational practice, positive organizational psychology, COVID-19

## Abstract

The COVID-19 pandemic has substantially impacted mental health—workers at institutions are not exempt. In our research, from positive organizational psychology, specifically from the healthy and resilient organization (HERO) model, we analyzed the relationship between healthy organizational practices–engagement and workers’ burnout, and evaluated the mediation role of engagement between healthy organizational practices and worker burnout levels during the COVID-19 pandemic, through structural equation models of a cross-sectional survey-based study. We collected data from a sample of 594 Chilean workers. Our results of the correlations and structural equations demonstrate the relationship between PHOs with engagement (β = 0.51; *p* < 0.001) and burnout (β = −0.44; *p* < 0.001), in addition to the mediating effect of engagement between HOP with burnout (β = −0.66; *p* < 0.001). In conclusion, our findings suggest that healthy organizational practices promoted worker engagement and decreased worker burnout during the COVID-19 pandemic, contributing to the postulates of the HERO model. In addition, we were able to visualize a similar scenario, which showed that burnout during a pandemic decreases when worker engagement mediates the relationship with HOP.

## 1. Introduction

Will COVID-19 have a substantial impact on the mental health of humanity? This is a question that Izaguirre-Torres and Siche [1] posed; positive answers are found in various articles [2,3,4,5,6]. They postulated that, irrespective of the condition (e.g., economic, social, cultural, etc.), we will all suffer from some type of psychological disorder.

Grover et al. [7] noted that, although only a group of people will be infected with COVID-19, and will have some physical health problems as a result, we will all have, more or less, mental health problems due to this pandemic.

The confinement/isolation of people has been the main measure taken by governments to stop the spread of COVID-19, which, according to Fiorillo et al. [8], has produced depressive symptoms, such as anxiety and stress.

Organizations and companies are not exempt. Salanova [9] noted that only some organizations will be able to “face changes proactively and grow with the crisis”. These are resilient and healthy organizations [10,11], companies that, through organizational practices and resources, have been promoting the well-being and health of their workers in order to generate positive work environments that prepare their workers to face the various events of life at these organizations, in a changing world.

Engagement is one variable that expresses well-being and health in workers [12]. In addition, workers with high levels of engagement show good individual and team performances [13,14,15,16,17,18,19], which impacts positive organizational results [11,20].

Various investigations show that managerial practices that organizations implement are fundamental to generate engagement in workers [21,22,23,24,25,26].

The healthy and resilient organization (HERO) model is a management model that proposes the development of planned–systematic practices and resources at organizations, which will generate positive work environments and have positive impacts on the well-being and health of employees; this in turn will promote good individual and team performances, implying healthy organizational results. The model proposes a group of healthy organizational practices (HOP). HOP influence worker engagement [23] as well as the generation of positive resources, such as the development of empathic teams [27], increasing organizational trust to improve the performance of workers [28].

The COVID-19 pandemic has led to (and will continue to lead to) mental health problems [29]. The following questions have arisen: can HOP influence the engagement and burnout of workers in pandemic times, just as these practices have done in times before COVID-19? Is engagement a mediating variable between HOP and worker burnout in times of a pandemic?

In this research, we analyzed the relationship between healthy organizational practices–engagement and workers’ burnout; we evaluated the mediation role of engagement between healthy organizational practices and worker burnout levels during the COVID-19 pandemic.

Although our approach was from positive organizational psychology, we could not extract ourselves from reality; therefore, we integrated burnout among the variables of this research due to its presence in workers during the COVID-19 pandemic [30,31,32].

## 2. Theoretical Framework

Since the beginning of the millennium, positive psychology has greatly developed in the organizational field [33,34] and in managerial perspectives [35], incorporating terms such as ‘healthy organizations’, gathering previous contributions since the 1960s [36,37,38]. This perspective argues that organizations must have positive and healthy employees to survive turbulent environments and non-manageable external situations—such as the current pandemic [10,11]. 

From this approach, organizations must develop management practices that promote good health and the well-being of their employees, which will imply good individual and team performances. Thus, these management practices will impact the financial health of the company and its results [20].

Using the HERO-based model [10], Acosta et al. [23] researched the relationship of healthy organizational practices (HOP) with work engagement. In a sample of 218 employees, the researchers developed correlations through Pearson’s coefficient and structural equations modeling. The nine practices of the model found positive and significant relationships (correlations from 0.16 to 0.39, *p* < 0.01). Furthermore, by applying the structural equation model, a positive and significant relationship between HOP and engagement (ß = 0.40, *p* < 0.001) was found.

Due to its relevance, the theoretical support in the HERO model, and its results, we defined the work of this research [23] as the initial model of our study, formulating the following hypothesis:

**Hypothesis** **0** **(H0).** *The relationship between the perception of healthy organizational practices and employee engagement was positive and significant during the COVID-19 pandemic.*

### 2.1. Healthy Organizational Practices

For Wright and McMahan [39] (p. 298), organizational practices are “a planned pattern of activities to facilitate an organization to achieve its goals”. They argue that those developed from human resources management are highly significant because they create a sense of belonging, commitment, and good performance among employees.

Research [21,23,40,41] has shown that the mere perceptions of employees on the existence of deliberate and systematic organizational practices, creates in them a sense of well-being.

Schaufeli et al. [42] conducted a one-year longitudinal survey with 201 employees. They found that organizational practices favoring autonomy, opportunities to learn, social support, and performance feedback correlated positively with vigor (*r* between 0.17 and 0.23, *p* < 0.01) and dedication (*r* between 0.28 and 0.34, *p* < 0.001), and negatively with cynicism (*r* between −0.25 and −0.30, *p* < 0.001). However, only social support correlated with burnout (*r* = −0.23, *p* < 0.001).

Matziani et al. [26] found that four types of organizational practices were positively and significantly related to engagement (*r* between 0.20 and 0.36, *p* < 0.001); and negatively and significantly related to burnout (*r* between −0.20 and −0.36, *p* < 0.001). However, when they applied linear regressions, they only found significant results when organizational practices predicted the dimension “dedication” (β = 0.31, *p* = 0.02).

Similar to research by Schaufeli et al. [42] and Matziani et al. [26], our research included employee burnout in the dependent variables and in engagement. Thus, we have formulated the following hypothesis that complements one of the initial models:

**Hypothesis** **1** **(H1).** *The relationship between the perception of healthy organizational practices and employee burnout was negative and significant during the COVID-19 pandemic.*

### 2.2. Engagement and Burnout

Engagement is a positive motivational state of vigor, dedication, and absorption [43], which is related to the way employees approach and cope with work, so it is not precisely a consequence. However, the type of management deployed can affect individuals and their state of engagement [44].

Burnout syndrome is a state of emotional exhaustion, cynicism, and low personal fulfillment [43,45]. Therefore, it is understandable that its consequences go beyond work and relate to other areas of employees’ lives.

Bakker et al. [46] note that research conducted on burnout has stimulated research on engagement, arguing that in the case of burnout, energy becomes exhaustion, involvement becomes cynicism, and effectiveness becomes ineffectiveness. They also highlight that burnout is expected to influence people’s functioning in the workplace and, therefore, their performance, similar to engagement, but positively, relating to job performance and organizational outcomes.

Parker, Bindl, and Strauss [47] reviewed various research studies. They concluded that employees with higher engagement tend to be more proactive, experience more significant learning, and have more creative behavior. Along the same line, Parker and Griffin [48] indicated that employees with higher engagement are more empowered and prepared to assume leadership in organizations. Nevertheless, the authors also argued that these people can be expected to be more prone to change in turbulent times and crises. 

Through applying structural equation modeling, Hussein [49] conducted a study on the engagement and burnout of 3786 employees. He found that higher engagement significantly reduced emotional exhaustion and depersonalization (β = −17.03 and −5.43, respectively, with *p* < 0.001). These results provided empirical evidence that a worker who experiences engagement with their job is less likely to present burnout [50,51] (Bakker et al., 2006; Cole et al., 2011). 

In the same way, but during the COVID-19 pandemic, Ahmed et al. [52] found that engaged employees with highly adaptive personality profiles showed less fear of COVID-19 and lower stress levels as better sleep qualities compared to the other personality profiles.

It is in this context that we have formulated our last hypothesis:

**Hypothesis** **2** **(H2).** *Engagement acts as a mediational variable for the perception of healthy organizational practices to decrease burnout.*

## 3. Materials and Methods

In this cross-sectional survey-based study, we carried out self-administered surveys through an online questionnaire between October and December 2020, after seven months of operation under confinement conditions in Ñuble, Chile (the first confined city to lockdown in Chile). Through convenience sampling in the stage, 594 surveys were obtained from different labor sectors: education (46.10%), health (20.02%), commerce (12.50%), industry (8.90%), and other items (12.30%).

In order to participate in the study, the workers had to be over 18 years of age, and, at the time of answering the questionnaire, formally work with signed contracts. Self-employed workers were not included.

The online questionnaire was sent via email to workers at different companies, after contacting representatives of the institutions, who delivered the database of workers who met the inclusion criteria.

Due to the conditions generated by the pandemic, it was not possible to contact all the companies in the Ñuble region, Chile.

### 3.1. Instruments

*Healthy organizational practices*. The statements proposed by the HERO model [10] were extracted from Acosta et al. [23]. Nine statements refer to: conciliation between work and family; prevention of mobbing; career development; skills development; occupational health; equity; social responsibility; communication; and information (e.g. “In this organization, mechanisms and strategies have been put in place over the last year to inform about the objectives of the organization so that they are known by all” see Appendix A). The statements were answered with a 7-point Likert-type scale. where 0 = “Never”; 1 = “Few times a year”; 2 = “Once a month or less”; 3 = “Few times a month”; 4 = “Once a week”; 5 = “Few times a week”; 6 = “Every day”. In the present study, adequate reliability was obtained (α*_HOP_* = 0.94).

*Engagement*. Engagement was evaluated with the Utrecht Work Engagement Scale (UWES [53]): vigor (6 items; for example, “When I get up in the morning, I feel like going to work”); dedication (5 items; for example, “I am enthusiastic about my work”), and absorption (6 items, for example, “I am happy when I am absorbed in my work”). All items were answered on a 7-point Likert scale that ranges from 0 (never) to 6 (always). In the current study, internal consistency was adequate in all dimensions (α*_Engagement_* = 0.90, α*_Vigor_* = 0.76, α*_Dedication_* = 0.84; α*_Absorption_* = 0.71).

*Burnout*. The Maslach Burnout Inventory General Survey (MBI-GS [54]) was used, adapted to the Spanish population [55], and was already used in Chilean samples [30]. This scale is made up of 15 items to measure the level of burnout on a frequency scale from 0 (never) to 6 (every day) points. As a whole, it provides a general burnout score, although it is usually analyzed according to the three classic dimensions reported in the literature: emotional exhaustion (5 items; for example, “I am emotionally exhausted from my work”); cynicism (4 items; for example, “I have lost interest in my work since I started in this position”), and professional inefficiency (6 items; for example, “In my opinion I am not good at my position”). In the present study, adequate reliability was obtained in all dimensions (α*_Burnout_* = 0.89, α*_Emotional exhaustion_* = 0.91, α*_Cynism_* = 0.85, α*_Professional inefficiency_* = 0.97).

### 3.2. Statistical Analysis

Reliability analyses and Pearson correlations between the dimensions of HOP with engagement and burnout were carried out; the eventual impact on engagement and burnout and the possible interactions between the last, were also evaluated using confirmatory models of structural equations. The confirmatory factor analysis was used as a means to build and evaluate theoretical models [56,57] and, thus, assess the explanatory capacities they had, as well as the HOP on the variables of engagement and burnout.

The estimation method used in this study was the one with maximum likelihood because it allowed recovering weak factors in the context of analyzing confirmatory models with *n* > 100 [57], at the same time, it allowed replicating the proposal by Acosta et al. [23] that was used as an initial model. The evaluation of the goodness-of-fit of a structural model was carried out by means of global fit indices of the model, using the chi^2^ statistic; the global fit indices (CFI) (which is interpreted as R^2^ in regressions and is valid in estimates using the maximum likelihood method, considering a good fit over 0.90); and the root mean square approximation error index (RMSEA), which allows analyzing the residuals, where the values that suppose a good fit in terms of residuals are those less than 0.08. In addition, the following indicators were used: comparative fit index (CFI), which corresponds to a standardization of the Bentler index and is recommended for use instead of chi^2^, together with RMSEA for samples over one hundred cases. A good fit is considered at values above 0.90; the non-normed fit index (NFI) or the Tucker–Lewis index (TLI), for which values equal to or greater than 0.90 are expected [58]. One indicator that allows a choice between alternative models is the parsimony normed fit index (PNFI), which ranges from 0 to 1, where higher values indicate a more parsimonious fit, even if the differences are less than 0.1 [58]. These last two indicators should be, at the discretion of Herrero [59], equal to or greater than 0.95. Therefore, the evaluation criteria for the goodness-of-fit of the model were RMSEA < 0.08; GFI, CFI, and TLI ≥ 0.95 [58,59].

All analyses were performed using JASP 0.16 software [60].

## 4. Results

The distribution by gender was 65.50% women and 34.50% men. According to age, 12.00% were under 24 years old, 30.60% were between 24 and 30 years old, 25.10% between 31 and 39 years old, 17.50% between 40 and 49 years old, 12.00% between 50 and 59 years old and 2.70% over 60 years old. Regarding the educational level, 2.00% corresponded to people with basic educational levels, 19.40% were people with complete middle educational levels, 44.90% studied at higher-level technical training centers, 30.80 % corresponded to people with professional studies, and 2.50% had postgraduate studies.

The means, standard deviations, and correlations between the study variables are presented in Table 1.

The correlations are as expected according to the literature reviewed, i.e., positive and significant for all healthy organizational practices (HOP) with engagement, and negative for all HOP with burnout. The effects regarding the correlations between HOP and engagement were medium and the correlations between HOP and burnout were small. Nevertheless, professional inefficiency has a poor or null correlation with HOP.

**Figure 1:**
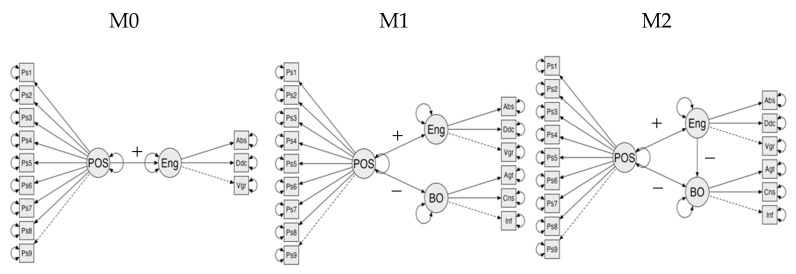
Specifications of hypothesized models of confirmatory structural equations. If there are multiple panels, they should be listed as: (**M0**) healthy organizational practices (HOP) impact engagement; (**M1**) HOP impact engagement and burnout separately; (**M2**) HOP impact engagement and burnout, and engagement mediates between HOP and burnout. Source: prepared by the authors.

As shown in Table 2, the models specificized in Figure 1, generally have adequate levels of adjustment, so they could all be accepted to understand the relationships between healthy organizational practices with the engagement and burnout variables (factor loading for each model can be found in Appendix B). Considering the parsimony index, the model that best explains the relationships between the variables is model 2, which presents an increase in the PNFI concerning model 0.

According to Table 3, firstly, the hypotheses H0 and H1 are accepted (and partially in H2) since the HOP → BO pathway was not significant. The effects of one variable on another were estimated using standardized path coefficients. Then, the magnitude of the indirect effects was estimated by multiplying the existing path coefficients along the casual line between two related variables. Thus, the indirect effect between HOP → engagement → burnout was −0.33. However, the initial model explains 25% of the variance in engagement, model 1 explains a similar proportion of engagement and 19% of the variance in burnout; and model 2, where engagement mediates between HOP and burnout, explains 47% of the variance in burnout.

## 5. Discussion

The objectives of this work were to analyze the relationship between healthy organizational practices with worker engagement and burnout and to study the mediation of engagement between healthy organizational practices and worker burnout during the COVID-19 pandemic. Recent research revealed the importance of work engagement for the well-being of workers and good performance [61,62]. Moreover, the COVID-19 pandemic increased employee burnout in organizations [63,64,65].

Although the hypothesized models present similar adjustments with acceptable solutions, we consider them advanced according to their developments concerning the existing literature, for the following reasons: model 0, which evaluates the impact of HOP on engagement, presents an explanatory capacity of the latter of 25%, which confirms hypothesis 0, although its explanatory level is lower compared to the 40% that is explained in its original study [23]. However, we consider the explanatory capacity model as adequate, considering that the conditions in which this sample corresponds to more significant uncertainty [66], a confinement situation [67], and restriction due to health decisions [68]. On the other hand, model 1 shows a similar variance to model 0, but has greater explanatory complexity when incorporating the impact of HOP on burnout, as stated in hypothesis 1, without affecting its ability to explain engagement. However, we believe that model 2 is the best because it relates the variables and what it allows, in terms of understanding the relationship between them. From our point of view, model 2 allows us to understand the joint impact and the mediating effect that engagement has between healthy organizational practices and burnout, confirming hypothesis 2. Moreover, it contributes to a deeper understanding of engagement as a variable orthogonal to burnout [30], as well as part of other personality variables that play moderating roles in models that use burnout as a negative outcome variable [69].

In general terms, these results support several recent investigations [70,71,72] that encourage the development of various management practices and resources to strengthen workers to face the consequences that the COVID-19 pandemic has brought to people’s work and personal routines. Thus, to face these types of consequences, healthy organizational practices are concrete ways to managing the mental health and well-being of workers. It is important to identify which healthy organizational practices are most closely related to the mental health and well-being of workers in order for planned and systematic efforts to take place at organizations. One of them is conciliation, which is relevant in this investigation, as well as in other documents, such as the Great Place to Work report. Our research provides evidence about this by working with two variables of broad interest in organizational behavior literature, helping to establish a relationship between them and their differentiations.

Among the limitations of the present study is that we did not include the influence of age and sex in the models analyzed. These two variables are relevant because evidence indicates that engagement is higher in women than in men and higher in people over 40 years old [30,44], which could explain differences in burnout differently. However, based on our initial model, we believe that the various impacts we experienced due to the COVID-19 pandemic should be analyzed in general, giving way to specific analyses according to various demographic variables, such as the gender and age of the participants, since these possible differences undoubtedly deserve to be treated exclusively in future research. Despite these limitations, the present work is a contribution because it complements and complicates the existing models in the literature [23] and, in turn, contributes to the intercultural validity of the HERO model [10], which allows us to understand that certain organizational practices are valid for different countries and issues relevant to workers today. It would be interesting to replicate this study in other Latin American countries and then apply it again sometime after the pandemic.

We consider it essential to point out that confirmatory analyses using structural equation models allowed us to explicitly specify the relationships between variables and evaluate their impacts on dependent variables, which contributed to the understanding of phenomena, such as the one studied here. However, and as indicated in the literature [73], we understand that structural equation models only allow providing (or not) support for the relationships of a hypothesized model. Therefore, we consider it essential to indicate that the inferences presented here should be interpreted with caution since different explanatory models could fit the empirical data equally well [74], inviting future research to deepen the work developed here.

## 6. Conclusions

Our findings suggest that healthy organizational practices are related to worker engagement and burnout during the COVID-19 pandemic, promoting engagement and reducing burnout, contributing to the postulates of the HERO model, developed in various investigations [10,23].

Our results allowed us to visualize a similar scenario, but in the same line, showing that burnout in times of a pandemic decreases when worker engagement mediates its relationship with healthy organizational practices.

These results strengthen the postulates of the scientific movement of positive psychology, especially those of positive organizational psychology. Positive organizational psychology establishes the relevance of the strengths of people (engagement specifically) and positive institution orientation (evaluated here as healthy organizational practices) so that people and organizations flourish and can face the various events of life (such as the COVID-19 pandemic) with proactivity and optimism, to survive them, and emerge stronger, seeing opportunities where others see catastrophe.

## Figures and Tables

**Table 1:** Correlations among the perceptions of healthy organizational practices (HOP), worker engagement, and burnout.

			Engagement						Burnout						Healthy Organizational Rractices															
	*M*	*SD*	1		2		3		4		5		6		7		8		9		10		11		12		13		14	
1. Absorption	4.72	0.95	—																											
2. Dedication	5.17	0.96	0.70	*	—																									
3. Vigor	5.09	0.76	0.70	*	0.75	*	—																							
4. Emotional exhaustion	2.47	1.49	−0.22	*	−0.36	*	−0.45	*	—																					
5. Cynicism	1.10	1.32	−0.37	*	−0.57	*	−0.54	*	0.57	*	—																			
6. Professional Inefficiency	3.38	2.24	−0.25	*	−0.17	*	−0.10	♺	0.08	♺	0.19	*	—																	
7. Reconciliation	3.72	1.77	0.28	*	0.36	*	0.34	*	−0.29	*	−0.22	*	−0.07		—															
8. Mobbing prevention	3.51	2.15	0.22	*	0.34	*	0.30	*	−0.24	*	−0.25	*	−0.07		0.65	*	—													
9. Skill development	3.77	1.85	0.24	*	0.35	*	0.29	*	−0.26	*	−0.22	*	−0.01		0.62	*	0.65	*	—											
10. Career Development	3.51	1.88	0.25	*	0.34	*	0.27	*	−0.22	*	−0.21	*	−0.03		0.58	*	0.56	*	0.75	*	—									
11. Labor Health	3.92	1.75	0.29	*	0.38	*	0.36	*	−0.32	*	−0.27	*	−0.08	♺	0.69	*	0.62	*	0.70	*	0.68	*	—							
12. Equity	3.28	1.98	0.29	*	0.34	*	0.36	*	−0.31	*	−0.22	*	−0.10	♺	0.58	*	0.53	*	0.67	*	0.63	*	0.69	*	—					
13. Information	4.05	1.74	0.30	*	0.38	*	0.36	*	−0.22	*	−0.23	*	−0.08		0.53	*	0.50	*	0.55	*	0.56	*	0.64	*	0.60	*	—			
14. Communication	4.23	1.66	0.33	*	0.40	*	0.37	*	−0.25	*	−0.21	*	−0.05		0.61	*	0.51	*	0.57	*	0.57	*	0.66	*	0.56	*	0.71	*	—	
15. CRS	3.82	1.83	0.31	*	0.43	*	0.36	*	−0.28	*	−0.27	*	−0.10	♺	0.58	*	0.60	*	0.65	*	0.66	*	0.71	*	0.63	*	0.67	*	0.68	*

* *p* < 0.001, ♺ *p* < 0.05; 1 = absorption; 2 = dedication; 3 = vigor; 4 = emotional exhaustion; 5 = cynicism; 6 = professional inefficiency; 7 = reconciliation; 8 = mobbing prevention; 9= skill development; 10= career development; 11= labor health; 12 = equity; 13 = information; 14 = communication; CRS = corporate social responsibility. Source: Prepared by the authors.

**Table 2:** Fit index for structural equation models.

Models	χ^2^	df	RMSEA	GFI	CFI	TLI	PNFI	Δχ^2^	Δ*df*	*p*
M0	274.34	53	0.08	0.92	0.96	0.94	0.759			
M1	447.15	87	0.10	0.88	0.91	0.89	0.748	172.81	34	<0.001
M2	614.82	88	0.08	0.91	0.94	0.92	0.763	167.67	1	<0.001

Source: prepared by the authors.

**Table 3:** Model path estimators.

	Model 0		Model 1		Model 2	
Predictor	*β*	*p*	*β*	*p*	*β*	*p*
HOP → Engagement	0.50	<0.001	0.51	<0.001	0.50	<0.001
HOP → BO			−0.44	<0.001	−0.04	0.42
Engagement → BO					−0.66	<0.001

Source: Prepared by the authors.

## Data Availability

Not applicable.

## Appendix A

Healthy organizational practices items

1. In this organization, mechanisms and strategies have been launched over the last year to facilitate the reconciliation of work life and private life of its employees
2. In this organization, mechanisms and strategies have been implemented over the last year to guarantee the prevention and management of mobbing
3. In this organization, mechanisms and strategies have been put in place over the last year to facilitate the development of workers’ skills
4. In this organization, mechanisms and strategies have been put in place over the last year to facilitate the career development of workers
5. In this organization, mechanisms and strategies have been implemented over the last year to ensure well-being and quality of life at work
6. In this organization, mechanisms and strategies have been put in place over the last year to guarantee that workers receive fair rewards in accordance with the effort we make
7. In this organization, mechanisms and strategies have been put in place over the last year to guarantee social responsibility issues in the organization
8. In this organization, mechanisms and strategies have been put in place over the last year to facilitate communication from the management to the workers, as well as from the workers to the management
9. In this organization, mechanisms and strategies have been put in place over the last year to inform about the objectives of the organization so that they are known by all.
